# Does the timing of special needs identification have an impact on children’s educational attainment, attendance, and exclusions?

**DOI:** 10.23889/ijpds.v9i5.2685

**Published:** 2024-09-18

**Authors:** Jennifer Keating, Alexandra Sandu, Katy Huxley, Rob French

**Affiliations:** 1 Cardiff University

Previous research has established an association between diagnosis of neurodevelopmental conditions and poorer academic outcomes. Many education policies advocate for early diagnosis and special education needs (SEN) support, however little is known about the association between timing of SEN identification and education outcomes.

To address this, we used Welsh administrative education data to identify the age at which a child first received SEN support. Using data from 90,271 children across 5 cohorts, logistic and linear regressions were run to examine the association between SEN identification and children’s attainment, exclusions, and attendance at age 16. In the latest cohort, we show that timing of SEN identification significantly associated with attainment, exclusions, and attendance. Children identified in their first year of school were 8 times less likely (than those without SEN) to meet national expectations for attainment, while those identified at age 16 were three times less likely, (X^2= 41.7, p<.001). The opposite pattern held true for exclusions and attendance: those identified in their final year were five times more likely to be excluded, compared to twice as likely for those identified in their first year (X^2= 7.3, p=.007). Those identified in final year were 2.52 times more likely to be persistently absent (missing >10% sessions), while those identified in their first year were 1.60 times more likely, (X^2= 8.9, p=.003). We show how these associations vary by gender, type of need, and timing of ending support. Early diagnosis is important across all outcomes, but most critical for attainment.

